# BiFeO_3_-Black TiO_2_ Composite as a Visible Light Active Photocatalyst for the Degradation of Methylene Blue

**DOI:** 10.1021/acsomega.3c00553

**Published:** 2023-05-15

**Authors:** Pravallika Banoth, Chinna Kandula, Praveen Kumar Lavudya, Saidulu Akaram, Luis De Los Santos Valladares, RajaniKanth Ammanabrolu, Ghanashyam Krishna Mamidipudi, Pratap Kollu

**Affiliations:** † School of Physics, University of Hyderabad, Prof. C. R. Rao Road, Gachibowli, Hyderabad, Telangana 500046, India; ‡ CASEST, School of Physics, University of Hyderabad, Prof C R Rao Road, Gachibowli, Hyderabad, Telangana 500046, India; § Cavendish Laboratory, Department of Physics, University of Cambridge, J. J. Thomson Avenue, Cambridge CB3 0HE, U.K.; ∥ Laboratorio de Cerámicos y Nanomateriales, Facultad de Ciencias Físicas, Universidad Nacional Mayor de San Marcos, Lima 14-0149, Peru

## Abstract

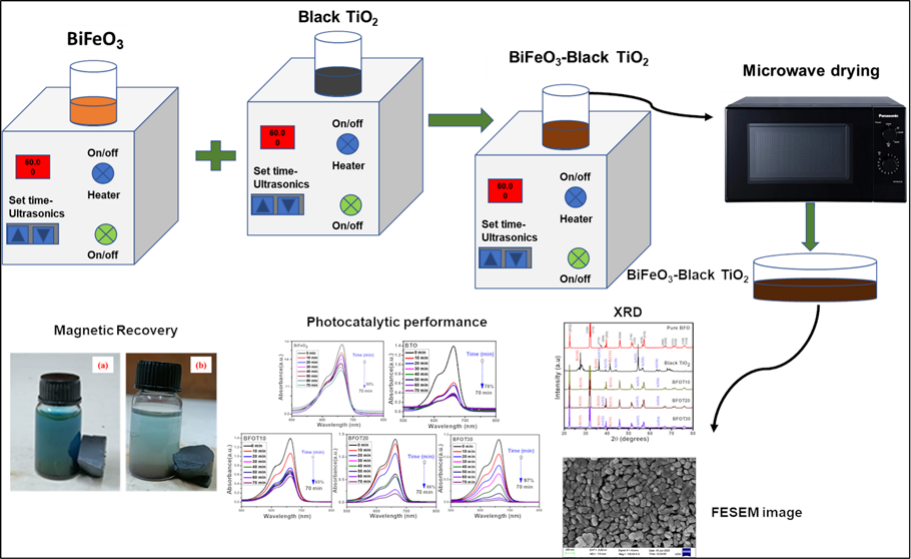

The application of a novel BiFeO_3_ (BFO)-black TiO_2_ (BTO) composite (called BFOT) as a photocatalyst for the degradation of methylene blue is reported. The p–n heterojunction photocatalyst was synthesized for the first time through microwave-assisted co-precipitation synthesis to change the molar ratio of BTO in BiFeO_3_ to increase the photocatalytic efficiency of the BiFeO_3_ photocatalyst. The UV–visible properties of p–n heterostructures showed excellent absorption of visible light and reduced electron–hole recombination properties compared to the pure-phase BFO. Photocatalytic studies on BFOT10, BFOT20, and BFOT30 have shown that they decompose methylene blue (MB) in sunlight better than pure-phase BFO in 70 min. The BFOT30 photocatalyst was the most effective at reducing MB when exposed to visible light (97%). Magnetic studies have shown that BTO is diamagnetic, and the BFOT10 photocatalyst exhibits a very weak antiferromagnetic behavior, whereas BFOT20 and BFO30 show diamagnetic behavior. This study confirms that the catalyst has poor stability and weak magnetic recovery properties due to the non-magnetic phase BTO in the BFO.

## 1 Introduction

A promising strategy to address environmental problems and the energy shortage is the use of catalysts for the remediation of organic pollutants.^1−6^ Photocatalysts are widely employed to break down contaminants using ultraviolet or visible light sources. Perovskite-based photocatalysts are a significant class of semiconductors that both UV and visible light can activate. Combining the photocatalysts is one method for improving their photoactivity because the efficiency of pure photocatalysts is frequently low.

A stable, non-toxic, and multiferroic visible light active photocatalyst has recently been explored to exist in the form of BiFeO_3_ (BFO). BFO has a band gap from 2.2 to 2.7 eV, which indicates that it strongly absorbs light in the visible range from 200 to 800 nm.^7,8^ Excellent characteristics of BFO have been seen in the degradation of pollutants. However, the main problem with the significant photogenerated electron–hole pairs recombination rate is still restricting the use of BFO for photocatalysis. To tackle this issue, BFO has received much attention when coupled with other visible active semiconductors, which prevents the high recombination of photogenerated electron–hole pairs and enhances photocatalytic activity.^9−13^

In recent years, black TiO_2_ functioned better as a photocatalyst than pure white TiO_2_ because it could better separate photogenerated electron–hole pairs. The higher solar light absorption of black TiO_2_ was caused by Ti^3+^ and the oxygen vacancies that resulted in refs (14)–^16^. Black TiO_2_ (BTO) photocatalysts have recently received a lot of interest due to their exceptional light-absorbing properties, which even make the NIR region of the solar spectrum practical. Due to the low band gap value, they showed light absorption even in the near-infrared range (1.54 eV).^17^ Since then, numerous successful photocatalysis and other photoelectrochemical applications have drawn significant research attention to BTO.^6^ Two usually mentioned benefits are increased photocatalytic efficiency and reduced recombination of photogenerated charge carriers. The heterostructure of multiferroic BFO and BTO nanoparticles is expected to be a superior photocatalyst with increased photocatalytic activity compared to pure-phase BFO.^18−21^

Furthermore, the shorter band gap of the heterostructure interfaces may result in more electrons and holes and exhibit higher photocatalytic efficiency.^22,23^ It is well known from the literature that recent studies on the BFO-based heterostructure have shown improved photocatalytic activity in organic dye degradation under visible light exposure. Therefore, creating strong photocatalytic activity based on black TiO_2_ seems to have potential.

To the present author’s knowledge, there are no reports on the composite of black TiO_2_ with BFO for organic dye degradation under visible-light exposure. In the current work, for the first time, BFO-BTO (black TiO_2_) heterostructures were synthesized through the microwave-co-precipitation method by mixing different molar ratios of BFO and the black TiO_2_ in the as-prepared condition by following microwave heating technique. The developed BFO-BTO composite (referred to by the acronym BFOT in the rest of the paper) photocatalysts with a narrow band gap have shown outstanding photocatalytic activity for methylene blue elimination under sunlight irradiation.

The novelty of this study is that it uses black TiO_2_ and multiferroic BiFeO_3_ to develop a bismuth-based composite for decomposing an organic dye in the visible range. For the photocatalysis application, there are no reports on this composite material. In previous works, bismuth-based composites made with white TiO_2_ had a low photodegradation percentage and took a long time to break down the dye completely. In our current study, however, bismuth-based composites made with black TiO_2_ showed a high photodegradation percentage in just 70 min of sunlight exposure.

## 2 Experimental Section

### 2.1 Materials Used for BFOT Heterostructures

For this procedure, ethanol, double-distilled water (DDW), pure potassium hydroxide (KOH, 98%), pure ferric chloride (FeCl_3_·6H_2_O, 98%), pure bismuth chloride (BiCl_3_, 98%), and commercial white TiO_2_ are all used.

#### 2.1.1 Preparation of BiFeO_3_ Micro Flowers

The pure-phase BFO microwave flowers are synthesized in 3 min at 800 W by microwave-assisted-solvothermal (MWAST) method in a domestic solo-microwave oven. The complete experimental procedure can be found in our previous work.^7^

#### 2.1.2 Preparation of Black TiO_2_ Nanoparticles

White TiO_2_ pellets were made utilizing simple but meticulous techniques. Ethylene glycol (EG) was added to the ultrafine TiO_2_ powder, which was then crushed for 60 min. EG was employed as a binder, and then, a pellet with the shape of a circular disc was formed by pressing a powder mixture with a hydraulic press at a pressure of 15 tonnes for roughly 2 min. The pellet is then sintered for 2–6 h at 800–1000 °C. Afterward, the white TiO_2_ pellets were irradiated by an electron beam for 40–60 min to get the black TiO_2_ [BTO] powder.

#### 2.1.3 Synthesis of (1 – *x*) BFO-TiO_2_ (*x* = 0.10, 0.20, and 0.30) Heterostructures

BFO-TiO_2_ heterostructures are prepared by a simple microwave-assisted-coprecipitation method following microwave heating at 360 W for 3 min. We dissolved a stoichiometric ratio of BFO and BTO powders in 100 mL of ethanol and sonicated it for 8 h at 80 °C to evaporate the solvent and obtain the gel. Finally, the gel was heated at 360 W for 3 min in a domestic microwave oven. Following the above procedure, we have synthesized three different heterostructures by loading different ratios of TiO_2_ onto the BFO. The prepared heterostructures (1 – *x*) BFO-*x*BTO, where *x* = 0.10, 0.20, and 0.30, are named BFOT10, BFOT20, and BFOT30, respectively.

### 2.2 Characterizations

UV–visible spectroscopy (Jasco V-670 UV–visible double beam spectrophotometer) was used to characterize the BFO, BTO, and BFOT heterostructures following synthesis, with ethanol serving as a blank. Wavelengths ranging from 200 to 800 nm were used to record the absorption spectra. Analysis of the X-ray diffraction (XRD) data (PANalytical, X’Pert Powder Diffractometer) has shown that the pure phases of BFO and BTO have been formed, as well as the creation of BFOT heterostructures. At a scan rate of 4 °/min and with a step size of 0.02°, the scanning range in this diffractogram was 20^θ^ to 80^θ^ [2θ value]. Field emission scanning electron microscopy (FESEM: Carl Zeiss Smart Sem) and transmission electron microscopy were used to study the morphology of synthesized samples. A vibrating sample magnetometer measured the magnetic properties of synthesized heterostructured samples at room temperature (VSM, Model: LakeShore).

### 2.3 Photocatalysis

Samples were tested for their photocatalytic ability to degrade methylene blue, an example of a pollution dye. A lot of sunlight was present during the photodegradation process. A total of 40 mg of each photocatalyst powder was initially added to a 100 mL MB solution at a concentration of 10 mg/L at a neutral pH. After the magnetic churning, the slurry was exposed to heavy sunlight. We used UV–visible spectroscopy to keep track of the dye solution’s decolorization.

## 3 Result and Discussion

### 3.1 X-ray Diffraction Analysis of BFOT Heterostructures

**Figure 1:**
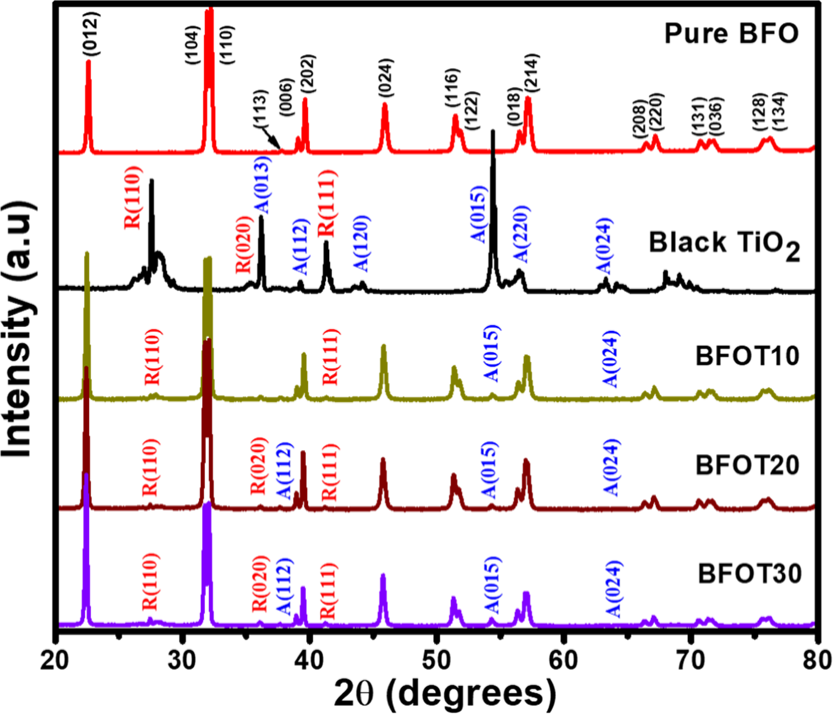
XRD patterns of pure BFO, BTO, BFOT10, BFOT20, and BFOT30.

In this study, BFOT heterostructure powders were synthesized through a microwave-assisted-chemical coprecipitation method and with BTO content levels of 10% (BFOT10), 20% (BFOT20), and 30% (BFOT30). The XRD patterns of BFO, BTO, and BFOT heterostructures are displayed in Figure 1.

The BFO displayed a Rhombohedral perovskite crystalline phase with the *R*_3*c*_ space group, which perfectly matched the prior crystallographic data (ICSD 98-019-1940) and showed no impurity phases within the detection range of the technique according to XRD examination. Tetragonal symmetry was found in the BTO XRD pattern, where anatase and rutile phases were present. The XRD patterns revealed all peaks belonging to pure-phase BFO with a low intense trace of the significant BTO peaks after adding 10% BTO (BFOT10). More intense BTO peaks began to form as the loading ratio of BTO reached 20% (BFOT20). After loading 30% BTO, the heterostructure sample BFOT30 finally displays all the high-intensity peaks from BTO and the diffraction pattern for pure BFO. The enhanced intensity of all the prominent BTO peaks besides the BFO peaks, increasing the BTO molar ratio up to 30%, confirms the higher BTO concentration.

### 3.2 FE-SEM Analysis of BFOT Heterostructures

**Figure 2:**
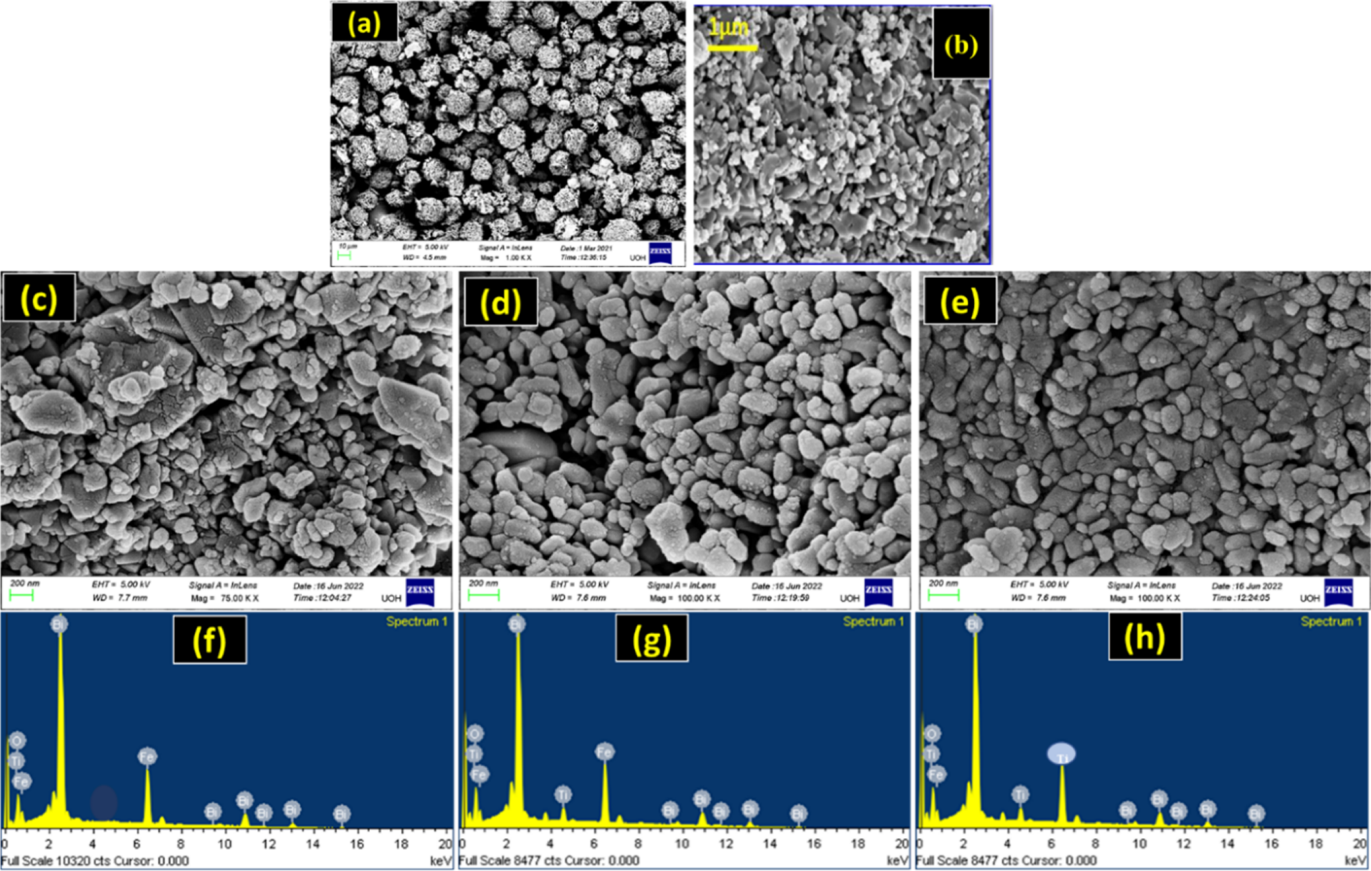
FE-SEM images of (a) pure BFO, (b) BTO, (c) BFOT10, (d) BFOT20, and (e) BFOT30 and EDAX spectra of (f) BFOT10, (g) BFOT20, and (h) BFOT30.

The various microstructures of the samples were examined using an FE-SEM, as shown in Figure 2. Figure 2a shows that the pure-phase BFO powder comprised many BFO micro flowers with hundreds of BFO nano petals packed tightly onto them.^24^ A considerable number of BTO nanoparticles of different sizes and shapes were formed, as demonstrated by the microstructures of pure-phase BTO in Figure 2b. Following the loading of BFO with various molar ratios of BTO particles, as shown in Figure 2c–e, the microstructure of the BFOT heterostructure is significantly changed. It is noteworthy to note that it is believed that BFO micro flowers were damaged after heating the BFOT heterostructures with high microwave energy. The BFOT10 heterostructure can be shown in Figure 2c to be made up of several grains of various sizes and fewer grain boundaries, which supports the hypothesis that the BFO and BTO phases are not as tightly coupled. According to Figure 2d, the contact between the two phases grows as the amount of BTO does, and the distribution of the grains is uniform with a uniform distribution of particle size. The microstructure of BFOT30 is finally formed of a large number of uniformly dispersed grains with an enormous number of grain boundaries, as seen in Figure 2e; this is because 30% BTO was added to the BFO. We can observe a stronger and more consistent coupling by comparing the BFO and BTO heterostructures to BFOT20 and BFT30 heterostructures. It is established from the EDAX spectra of BFOT10, BFOT20, and BFOT30 in Figure 2f–h that the Bi, Fe, O, and Ti components are the source of the signal peaks, demonstrating that BTO was successfully loaded onto the BFO.

### 3.3 Optical Properties of BFOT Heterostructures

**Figure 3:**
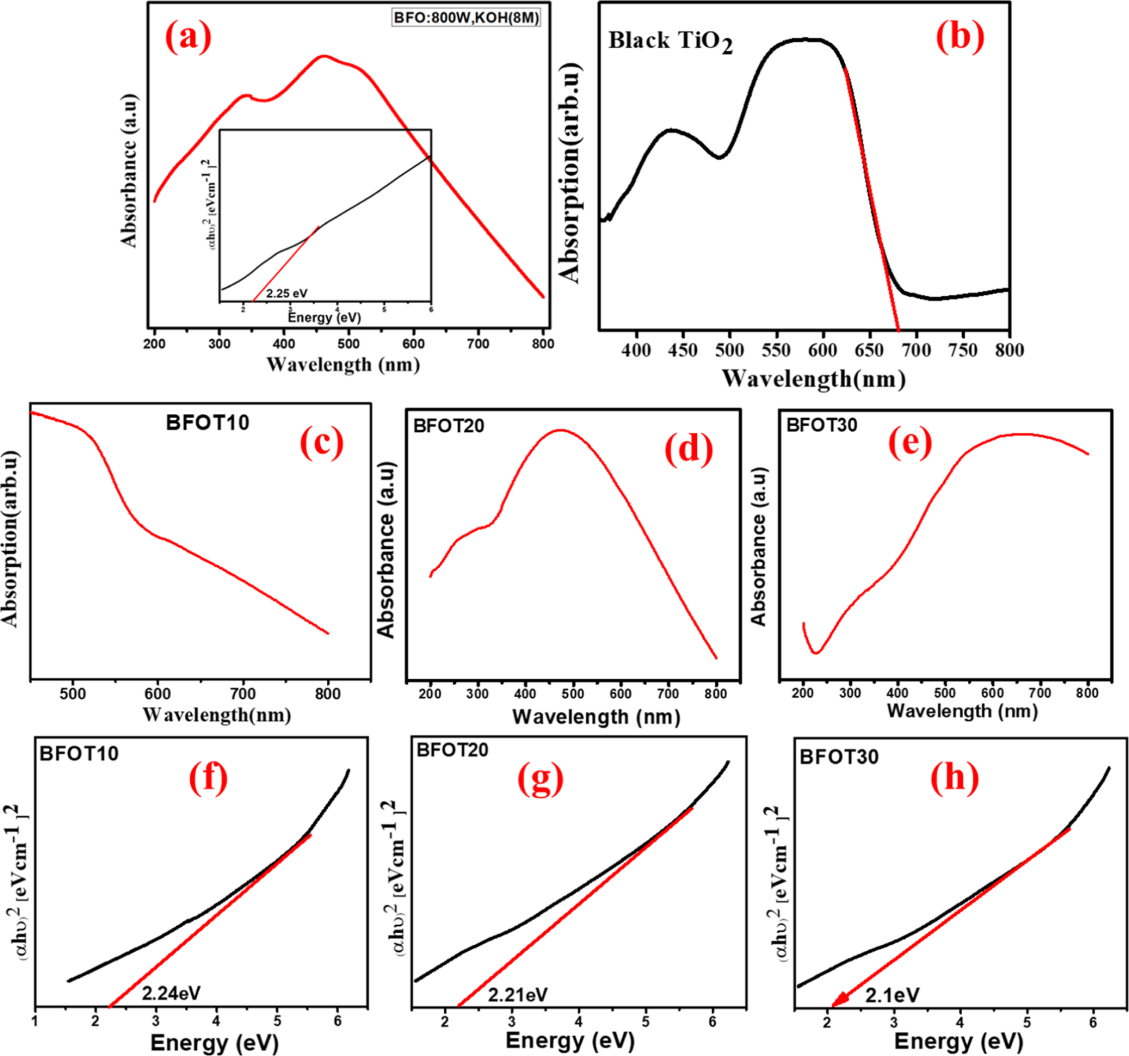
UV–vis absorption spectroscopy of (a) BFO (band gap in the inset), (b) BTO, (c) BFOT10, (d) BFOT20, (e) BFOT30, and energy band gap of (f) BFOT10, (g) BFOT20, and (h) BFOT30.

The UV–vis absorption spectrum of all the prepared samples is shown in Figure 3a–h. Each sample demonstrated excellent visible light absorption. Figure 3a,b and the inset inside Figure 3a depict the light absorption and energy band gap of the pure phases of BFO and BTO. A pure-phase BFO exhibits light absorption at 550 nm with a band gap of 2.25 eV (Figure 3a and inset, respectively). In contrast, a pure-phase BTO exhibits light absorption at 690 nm with a band gap of 1.68 eV (Figure 3a and inset, respectively). The UV–vis absorption spectrum of all three BFO-BTO heterostructures is shown in Figure 3c–e. From these light absorption properties, we can see that BFOT10 is absorbing light at the 552 nm range (Figure 3c), BFOT20 is absorbing light at the 560 nm range (Figure 3d), and BFOT30 is showing strong absorption in the higher wavelength side, i.e., at 650 nm (Figure 3e). It is essential to discuss that with the increase in the BTO content in the BFO, the light absorption properties of the composite materials in the visible range have increased, i.e., BFOT10 showing absorption at 552 nm, BFOT20 at 560 nm, and BFOT30 at 650 nm, therefore shifting of the band absorption toward the higher wavelength side indicates the increase in the light absorption property in the visible range, so this enhancement in the light absorption properties leads the production of higher no of electrons and holes in the composite materials.^25−30^ Therefore, from this UV–vis spectral studies, all the heterostructures BFOT10, BFOT20, and BFOT30 have shown excellent optical absorption in the visible range, confirming the capability of these heterostructures for the photodegradation of organic dyes in the visible range.

The following formula determines the band gap of the heterostructures.
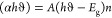
α is the absorption coefficient, *E*_g_ represents the energy band gap, *A* is constant and depending on transition, we have to consider *n* values, direct permitted transitions, *n* = 1/2, direct forbidden transitions, *n* = 3/2. Permitted indirect transitions, *n* = 2. Indirect prohibited transitions, *n* = 3.

The obtained band energies of these heterostructures BFOT10 (552 nm), BFOT20 (560 nm), and BFOT30 (650 nm) are shown in Figure 3f–h, and the obtained band gap values were 2.24 (Figure 3f), 2.21 (Figure 3g), and 1.91 eV (Figure 3h). The obtained band gap studies show a reduction in the energy band gap of these heterostructures compared to the pure-phase BFO, which could be due to the combination of two different energy band gaps at the interface between two semiconductors.^31^ As a result, BFOT30 was the most efficient photocatalyst when absorbing visible-light photons among the coupled photocatalysts. Optical energy band gaps obtained from UV–vis absorption studies are shown in Table 1.

**Table 1:** Optical Energy Band Gaps Obtained from UV–Vis Absorption Studies

s. no	sample	the band gap (eV)
1	BFO (Figure 4)^15^	2.25
2	BTO	1.68
3	BFOT10	2.24
4	BFOT20	2.21
5	BFOT30	2.1

### 3.4 Brunauer–Emmett–Teller Analysis of BFOT Heterostructures

**Figure 4:**
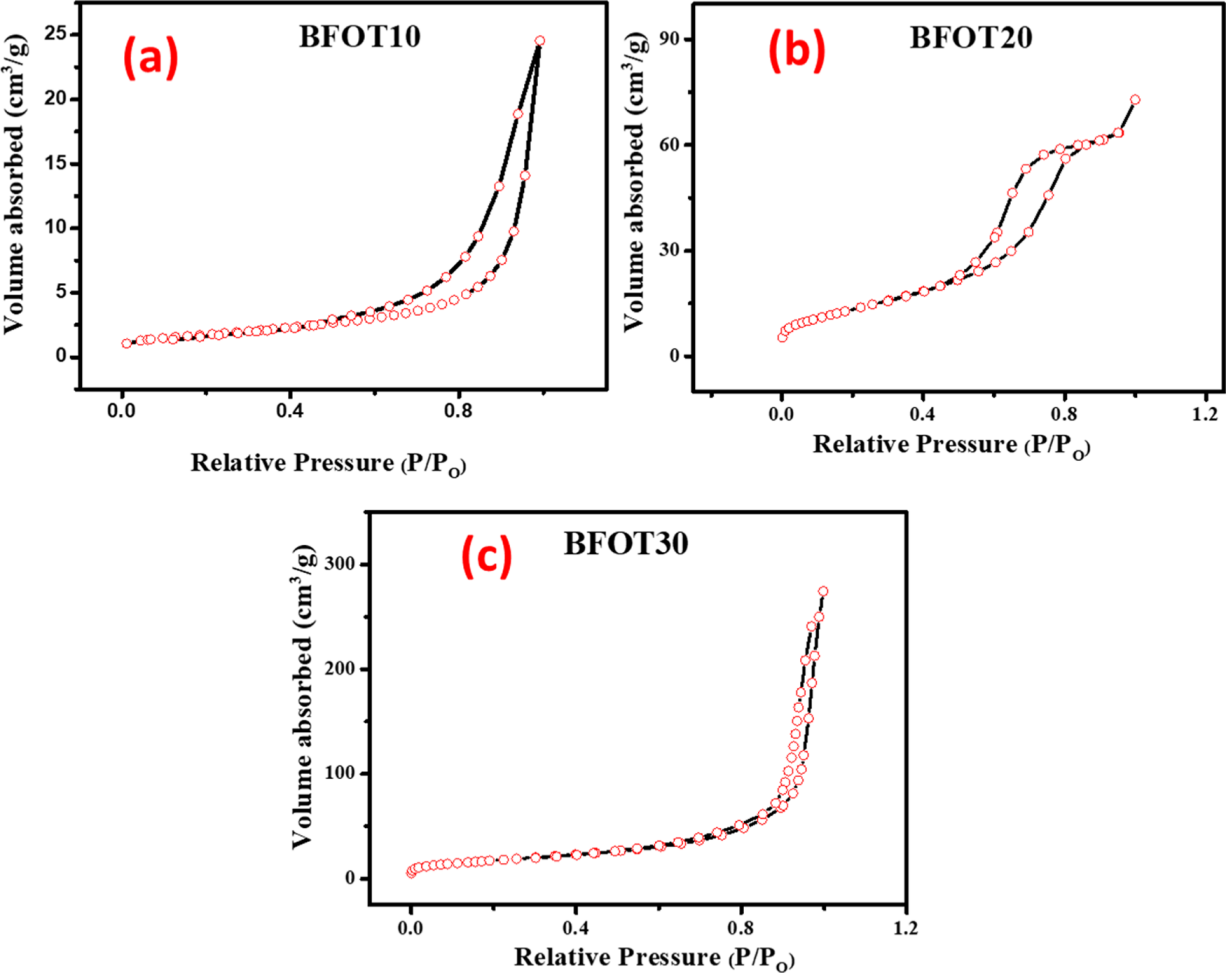
Nitrogen absorption/desorption isotherms of (a) BFOT10, (b) BFOT20, and (c) BFOT30.

Nitrogen adsorption–desorption isotherms were used to study the specific surface areas of the BFOT samples made through microwave-assisted. Figure 4a–c shows the Brunauer–Emmett–Teller (BET) surface area curves of the BFOT heterostructure nanoparticles made using microwave-assisted co-precipitation. All of the BFOT samples BFOT10, BFOT20, and BFOT30 that were made had a type IV nitrogen isotherm with a hysteresis loop. The shape of the hysteresis loops for the BFOT10, BFOT20, and BFOT30 samples was all H3 type.^32^ The obtained specific surface areas of the samples BFOT10, BFOT20, and BFOT30 are 46.10, 48.98, and 54.77 m^2^/g, respectively. The higher specific surface area sample BFOT30 can enhance the degradation efficiency.

### 3.5 Photocatalytic Activity of BFOT Heterostructures

**Figure 5:**
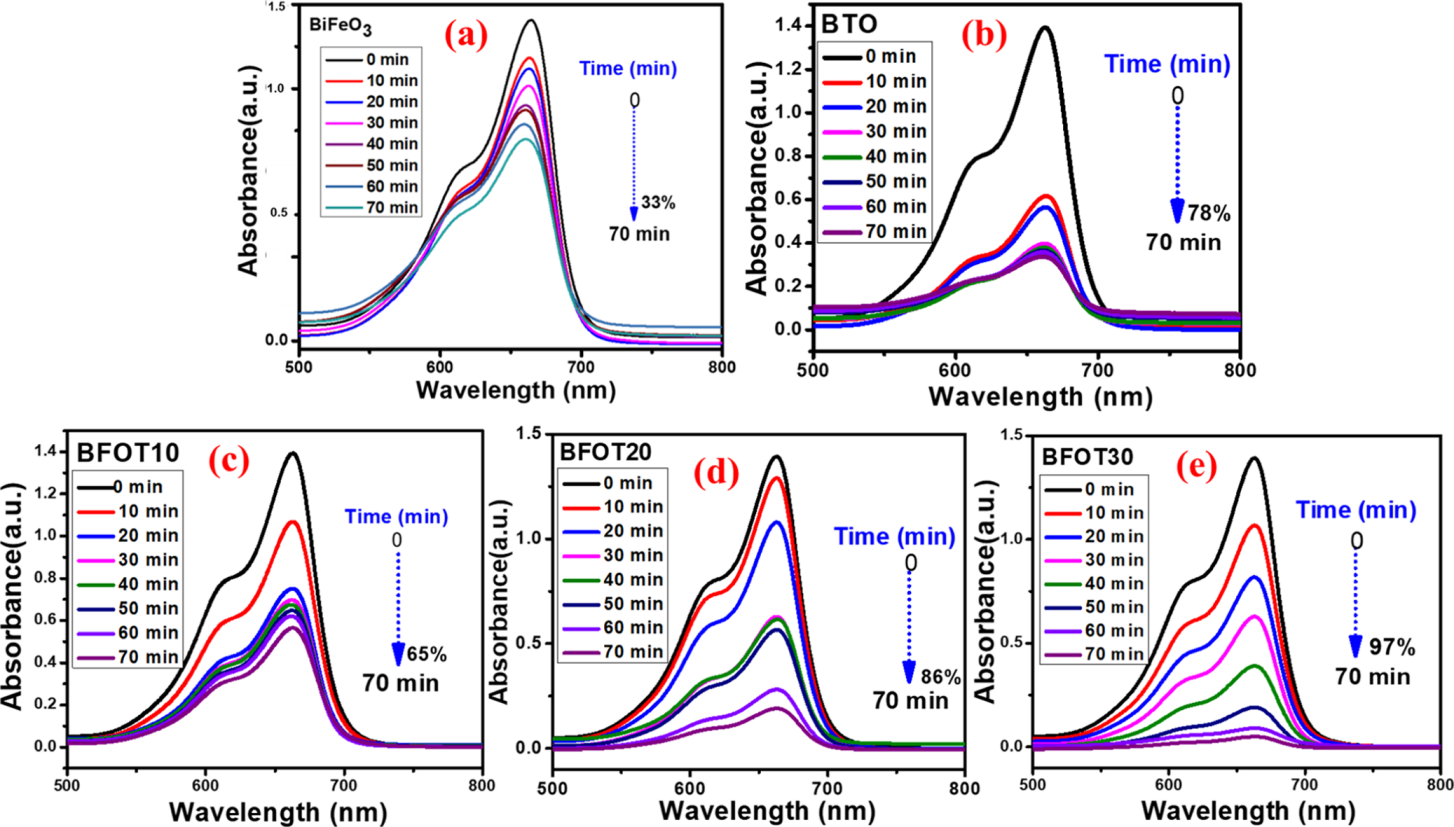
Time-dependant UV–vis spectral changes of MB in the presence of (a) BFO, (b) BTO, (c) BFOT10, (d) BFOT20, and (e) BFOT30.

Figure 5a–e shows the photocatalytic activity of pure-phase BFO, pure-phase BTO, and BFOT heterostructures. The total degradation of MB for the BFO, BTO, and BFOT heterostructures took 70 min to complete. As shown in Figure 5a, the pure-phase BFO is showing a poor photodegradation efficiency of 33% and a pure-phase BTO is exhibiting 78% degradation in MB (Figure 5b), which is higher photodegradation efficiency compared to the pure-phase BFO. Figure 5c–e depicts the photocatalytic performance of BFO-BTO heterostructures. After adding 10% BTO into the BFO [BFOT10], we observed less degradation (65%) in the MB solution in the presence of the BFOT10 catalyst (Figure 5c) when compared to the other two heterostructures [BFOT20 and BFOT30]. From Figure 5d,e, we can see that the efficiency of the catalysts was increased by increasing the BTO content into the BFO, where the BFOT20 (86%) and BFOT30 (97%) catalysts exhibited enhanced degradation in MB after 70 min of sunlight illumination, as shown in Figure 5d,e, respectively. The increased photocatalytic performance of the samples BFOT20 and BFOT30 demonstrate how efficiency increased for higher concentrations of BTO. The higher photodegradation efficiency (97%) of the BFOT30 heterostructure is due to its microstructures and more significant specific surface-to-volume ratio than the BFOT10 and BFOT20 heterostructures. The other reason for higher photodegradation efficiency is that BFOT30 has a narrower band gap than the other two samples, making it easier for light to form more electrons and holes at the surface of BFOT. As a result, its efficiency is improved.^33^ This photodegradation test demonstrates excellent results for the rapid photodegradation efficiency of heterostructures compared to pure-phase BFO, demonstrating these heterostructures’ abilities to degrade MB in sunlight. Photocatalyst performance is determined by the equation below. Both *C*_0_ and *C*_*t*_ represent MB’s initial and time-corresponding concentrations, respectively.
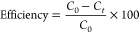


### 3.6 Effect of Photocatalyst Concentration on Photocatalytic Efficiency

**Figure 6:**
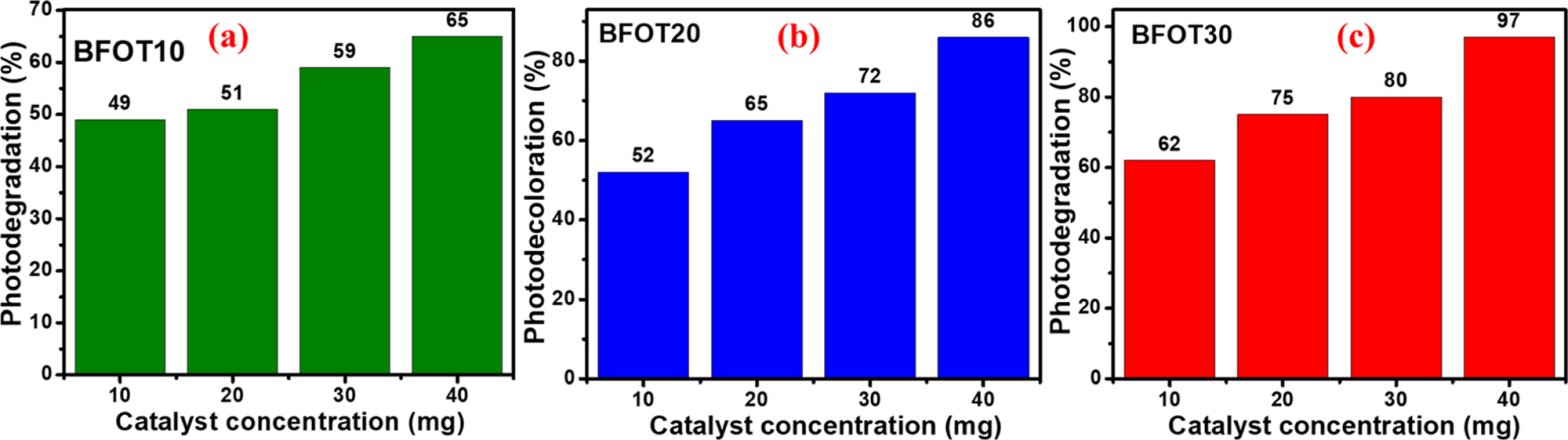
Effect of photocatalyst dosage on the photodegradation efficiency of heterostructures (a) BFOT10, (b) BFOT20, and (c) BFOT30.

Different heterostructures with BFOT10, BFOT20, and BFOT30 catalyst doses were used to degrade the MB dye at a constant dosage of 10 mg/L (10, 20, 30, and 40 mg are shown in Figure 6a–c). From Figure 6a, we can see that as increasing the catalyst BFOT10 concentration from 10 to 40 mg, the photodegradation percentage increased from 49 to 65%, similarly for BFOT20, photodegradation increased from 52 to 86% (Figure 6b), and for BFOT30 increased from 62 to 97% (Figure 6c) (all the obtained values are shown in Table 2), this analysis is showing that the photodegradation efficiency is substantially dependent on the catalyst concentration. The literature well established that the microstructures of the catalysts and their micro-dosing, starting dye dose, type, and pH value all significantly impact the photodegradation efficiency.^34−38^

**Table 2:** Influence of Catalyst Dosage (mg) on Photodegradation (%)

dye Name	catalyst name	catalyst dosage (mg)	time (min)	degradation (%)
MB	BFOT10	10	70	49
		20	70	51
		30	70	59
		40	70	65
MB	BFOT20	10	70	52
		20	70	65
		30	70	72
		40	70	86
MB	BFOT30	10	70	62
		20	70	75
		30	70	80
		40	70	97

### 3.7 Room-Temperature Magnetic Properties

**Figure 7:**
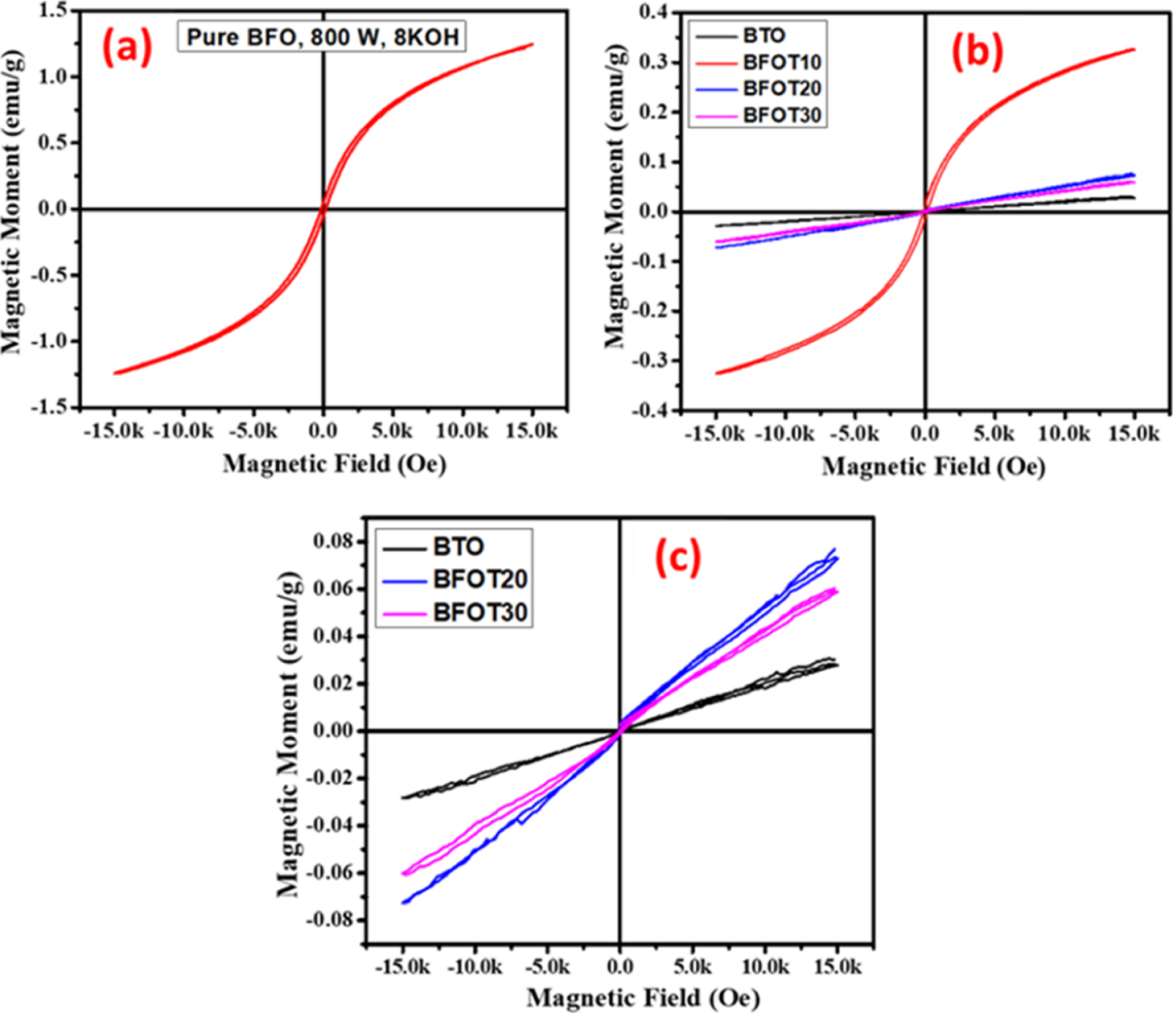
Magnetic properties of (a) BFO, (b) BFOT heterostructure, (c) zoomed M–H loops for BTO, BFOT20, and BFOF30.

The room-temperature magnetic properties of BTO and BFOT heterostructures are shown in Figure 7. Figure 7a,b shows that single-phase BFO has a high magnetization value of 1.25 emu/g, while black TiO_2_ is diamagnetic when a magnetic field is applied. The magnetic properties of BFOT have decreased with an increase in the diamagnetic phase BTO loaded on weak ferromagnetic phase BFO (Figure 7b). When 10% diamagnetic BTO is added to BFO, the magnetization value is reduced to 0.32 emu/g (BFOT10). After increasing the BTO molar ratio to 20 and 30%, the heterostructures BFOT20 and BFOT30 show linear M–H curves, indicating they are diamagnetic [(Figure 7c) shows the close-ups of the M–H loops for BTO, BFOT20, and BFOT30].

### 3.8 Stability and Reusability Test for BFOT Photocatalysts

**Figure 8:**
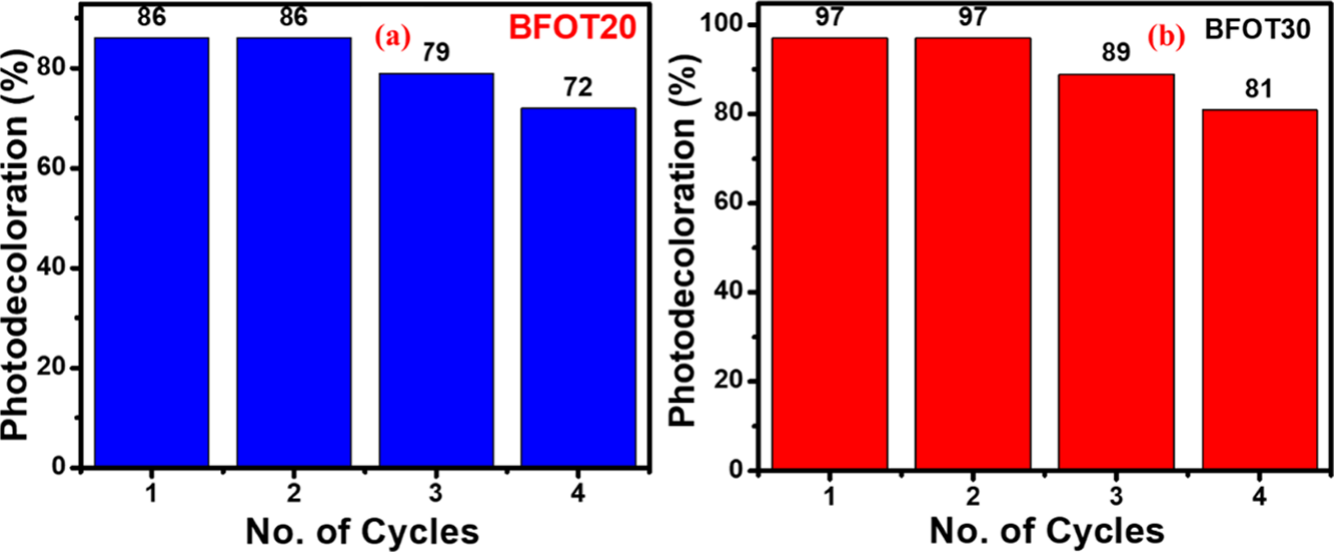
Photodegradation efficiency of heterostructures (a) BFOT20 and (b) BFOT30 after four cycles.

The stability of catalysts was investigated by recycling the most productive powders, BFOT20 and BFOT30 catalysts, four times, as shown in Figure 8a,b. According to Figure 8a, there is no difference in the photodegradation efficiencies of the BFOT20 catalysts for the first two cycles, but the catalysts’ poor stability is demonstrated by a total 13% decline in BFOT20’s photodegradation efficiency after two cycles. Similarly, for BFOT30 catalysts, there is no difference in the photodegradation efficiencies for the first two cycles, but the catalysts’ poor stability is demonstrated by a total 16% decline in BFOT20’s photodegradation efficiency after two cycles (Figure 8b).

**Figure 9:**
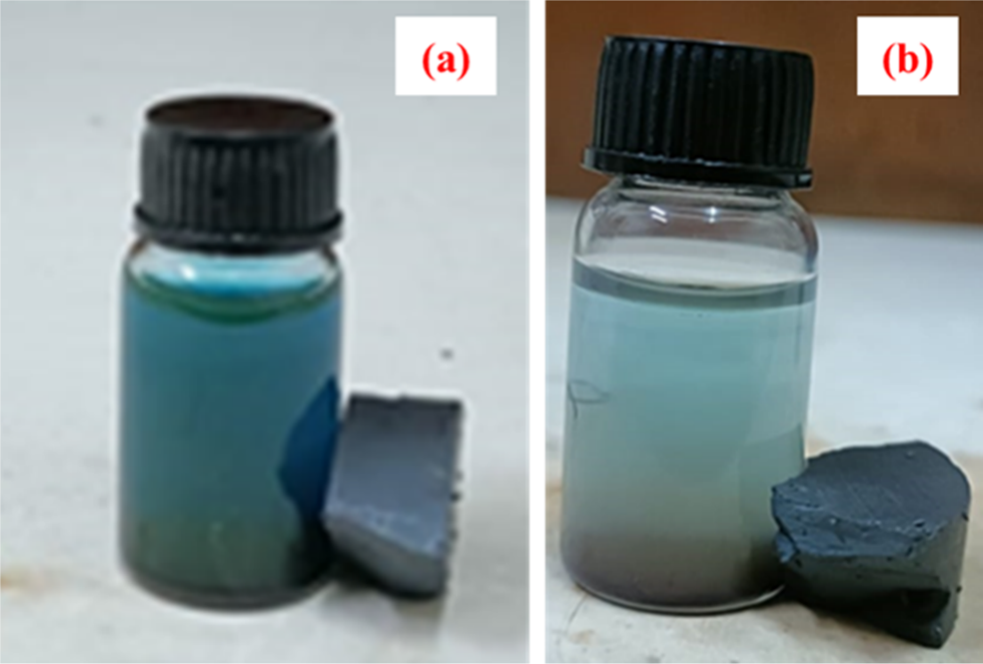
Magnetic recovery test for (a) BFO and (b) BFOT30.

Figure 9a highlights how pure-phase BFO magnetic responses fail to recover particles when applied to magnetic fields after the photodegradation experiment. Therefore, additional filtering or centrifugation is required to separate the catalyst from the MB solution.^25−30^ To study the magnetic recovery of the most effective photocatalyst, BFOT30, we applied the magnetic field shown in Figure 9b. Figure 9b shows that BFOT30 cannot be recovered when a magnetic field is applied due to its diamagnetism, confirming its weak magnetic recovery. The results of these stability and recovery studies indicate that the stability and magnetic recovery properties of BFO did not improve, even when black TiO_2_ was added. When a magnetic field was applied to BFOT, the magnetic response may have been reduced and exhibited diamagnetic properties due to the non-magnetic TiO_2_ loaded on weak magnetic phase BFO.

### 3.9 XRD and FESEM Analysis of the Most Active BFOT30 Catalyst after the Photocatalytic Test

**Figure 10:**
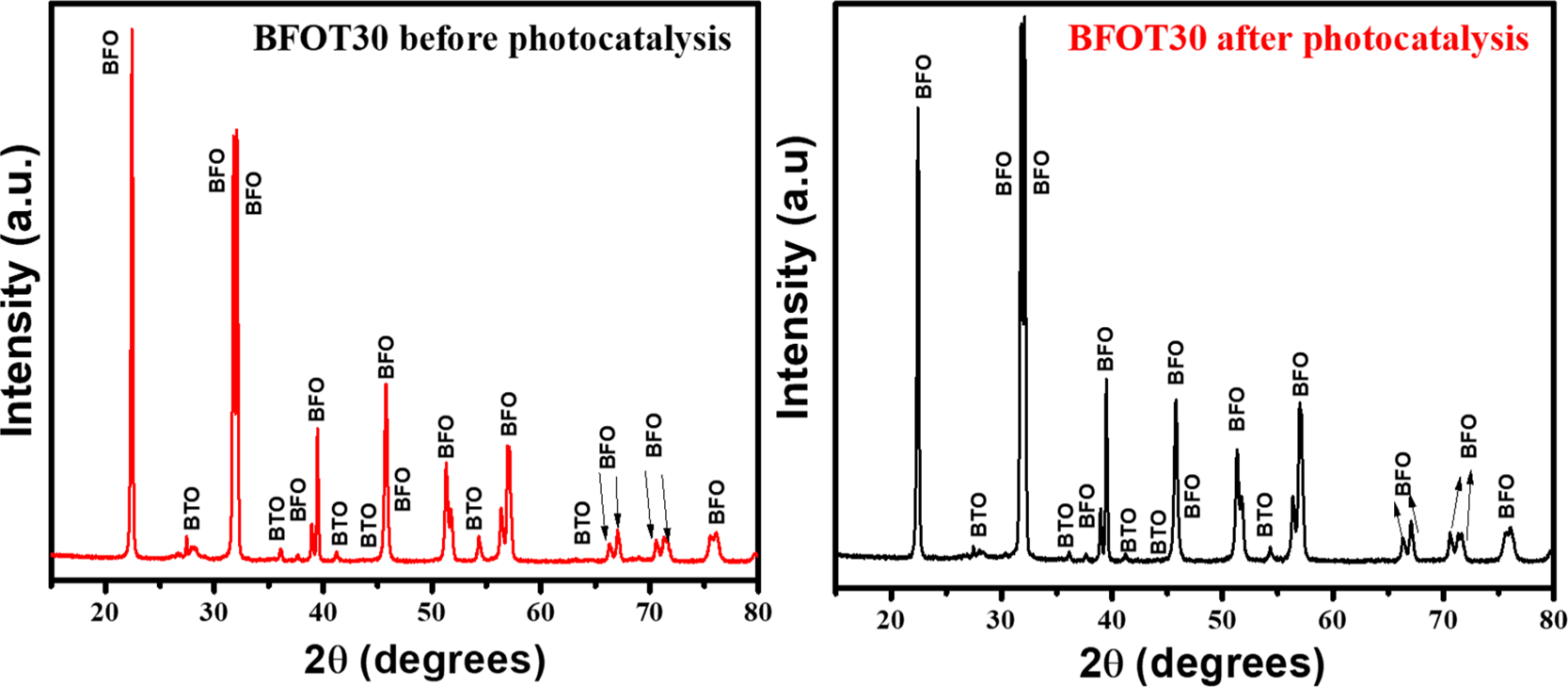
XRD patterns of a BFOT30 heterostructure before and after the photocatalysis test.

The BFOF30 powder underwent XRD and FESEM analysis to see if it could be appropriately recovered or not at the beginning and end of the photocatalytic process and recovery test. The XRD patterns for BFOF30 before and after MB degradation are shown in Figure 10. We discovered no differences in the XRD patterns or traces from MB for the BFOF30 catalyst used in the photocatalysis experiment. Therefore, BFOF30 catalysts that are centrifugally recovered retain their original structural characteristics.

**Figure 11:**
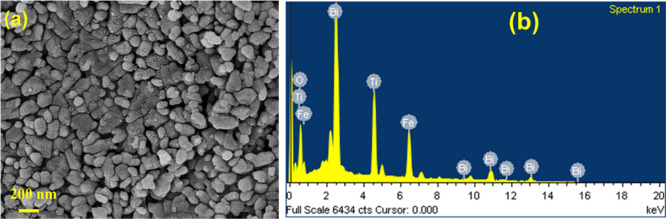
FESEM image (a) and EDAX spectra (b) of a BFOT30 heterostructure after the photocatalysis test.

The FE-SEM image and EDAX of the BFOF30 catalyst after four cycles are shown in Figure 11a,b. We can see from the microstructural analysis of the BFOT30 sample that it was composed of many grains and boundaries without any agglomerations and other structures, confirming that no microstructures correspond to MB Figure 11a. We have observed that the BFOT30 catalyst’s microstructures are similar before and after the photocatalysis and recovery test, indicating the stability of this sample’s microstructural characteristics even after four cycles. Figure 11b shows the EDAX spectra of the BFOT30 catalyst after photocatalysis and recovery test; from these EDAX spectra, it is observed that the Bi, Fe, O, and Ti components are the source of the signal peaks, and no other peaks corresponding to MB was found demonstrating that BFOF30 catalysts that are centrifugally recovered retain their original microstructural characteristics.

## 4 Conclusions

The BFOT heterostructure was successfully processed by coprecipitation with the help of microwave for MB degradation. Microstructure analysis and UV–vis absorption spectroscopy in visible light have shown that forming nano-interfaces between the BFO and black TiO_2_ phases can change the band gap and encourage changes in the work function at the surface of the heterostructures, improving the creation of electron–hole pairs and impeding its recombination. After 70 min in the sun, BFOT produces the best (97%) results, with the highest deterioration in MB. The photocatalyst can only be utilized for two cycles with a 16% reduction in efficiency, according to the recycling test for the most effective BFOT30. It also cannot be retrieved using a magnetic field. A photocatalytic test showed that black TiO_2_ increased the activity of pure-phase BFO when added to it. According to our findings, a simultaneous electron drain process between BFO and BTO and a direct Fenton-like mechanism contributed to the BFOT heterostructure’s enhanced photocatalytic efficiency.
